# Innovative Art-based Interventions Designed to Reduce Stress and Enhance Coping Strategies

**DOI:** 10.1192/j.eurpsy.2023.2097

**Published:** 2023-07-19

**Authors:** D. Segal-Engelchin, V. Daichman, O. Sarid

**Affiliations:** 1 Ben-Gurion University of the Negev, Beer Sheva, Israel

## Abstract

**Introduction:**

Short term and immediate interventions are an essential tool to combat distress in times of community crises. The combination of cognitive behavioral interventions and art practice provides a unique tool for the transformation of stressful visual images into less threatening and more manageable images. Previous research has demonstrated the efficacy of cognitive behavioral- and art-based (CB-ART) interventions in reducing distress related to different types of community crises.

**Objectives:**

The aims of the current study were (1) to compare the effectiveness of CB-ART interventions in reducing distress associated with two types of ongoing community crises: actual war conditions and the Covid-19 pandemic; and (2) to compare the mechanisms used in these contexts to transform the stressful image associated with the community crisis into a more manageable image.

**Methods:**

CB-ART workshops were conducted during both the 2014 Israel–Gaza conflict and the first wave of COVID-19 in Israel. The CB-ART workshops included drawing pictures related to three topics: (1) emotions and thoughts related to the ongoing community crisis; (2) coping resources; and (3) integration of the stressful image and the resource picture. To examine the intervention effect, the Subjective Units of Distress (SUDs) values of the two *affected g*roups were measured using a pre-post design.

**Results:**

In both groups participants’ distress levels significantly decreased after the intervention. A significantly larger decrease was found among the group that participated in the CB-ART workshops during war conditions. The dominant compositional changes within the integrative picture that emerged in both groups included a diminished size of the stressful image; use of several mixed-sized objects scattered all over the drawing, as opposed to one large–sized object placed at the center of the drawing, which typically characterized the stress drawing; and use of lighter optimistic colors.

**Conclusions:**

Similar mechanisms of alterations in the stressful image were found in the two groups, accompanied by reduced distress, as depicted by the SUDs values reported by the participants. Future studies would benefit from examining the effectiveness CB-ART interventions in reducing stress and enhancing coping strategies during additional community crises.

**Disclosure of Interest:**

None Declared

